# Subcutaneous ticks: a first report in a golden jackal, and their absence in non-canid carnivores

**DOI:** 10.1186/s13071-020-04510-7

**Published:** 2021-01-05

**Authors:** Noureddine Mechouk, Georgiana Deak, Angela Monica Ionică, Dan Traian Ionescu, Gabriel Bogdan Chișamera, Călin Mircea Gherman, Andrei Daniel Mihalca

**Affiliations:** 1 Ecology of Terrestrial and Aquatics Systems Laboratory (EcoSTAq), Department of Biology, Faculty of Science, Badji Mokhtar University—Annaba, Annaba, BP 12, 23200 Algeria; 2 Department of Parasitology and Parasitic Diseases, Faculty of Veterinary Medicine, University of Agricultural Sciences and Veterinary Medicine of Cluj-Napoca, Calea Mănăștur 3-5, Cluj–Napoca, 400372 Romania; 3 Molecular Biology and Veterinary Parasitology Unit (CDS 9), Regele Mihai I al României Life Science Institute, University of Agricultural Sciences and Veterinary Medicine of Cluj-Napoca, Calea Mănăștur 3-5, 400372 Cluj-Napoca, Romania; 4 Department of Game and Wildlife, Faculty of Silviculture and Forestry Engineering, Transilvania University, Şirul Beethoven 1, 500123 Braşov, Romania; 5 Grigore Antipa National Museum of Natural History, Sos. Kiseleff no. 1, 011341 Bucharest 1, Romania

**Keywords:** Golden jackals, Subcutaneous, Ticks, Romania

## Abstract

**Background:**

Ticks are hematophagous arthropods which normally attach to the surface of the host’s skin. Their aberrant presence in the subcutaneous tissue of a few carnivores, predominantly foxes, has been reported. However, there have been no reports of this phenomenon in other carnivores such as mustelids or golden jackals. Our aim was to investigate the host spectrum for this aberrant localization of ticks.

**Methods:**

Between 2015 and 2020, a total of 198 carcasses of 12 species of carnivore were examined by parasitological necropsy. When a subcutaneous tick was found, the nodule was removed, carefully dissected, and stored in ethanol. The morphological identification of the subcutaneous tick was carried out to species level.

**Results:**

A single subcutaneous tick was found in one carcass, that of a golden jackal (*Canis aureus*). The tick was identified as a female *Ixodes ricinus*. All the other carcasses were negative for the presence of subcutaneous ticks.

**Conclusion:**

To our knowledge, this is the first report of a subcutaneous tick in a golden jackal. This finding broadens the host spectrum of subcutaneous ticks, and reinforces the idea that, among carnivores, this phenomenon only occurs in canids. 
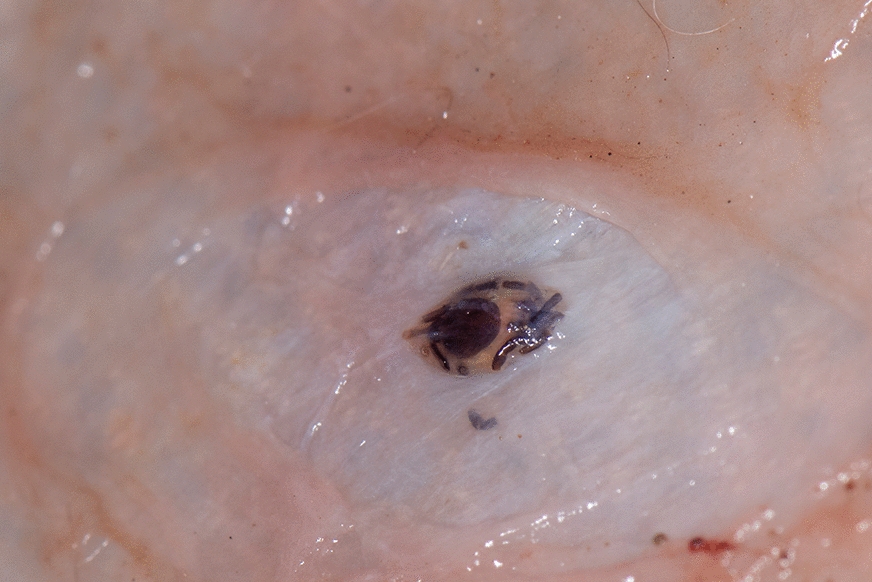

## Background

Ticks represent a large group of blood-sucking arthropods and are parasitic in a wide range of mammals, birds, reptiles, and occasionally amphibians. Ticks are also important vectors for various pathogens [1]. In ticks, a blood meal is required for molting, egg laying, and, in some species, preparation for mating [2].

Ticks typically attach to the external surface of the skin. However, there are reports of ticks being found in subcutaneous tissue (Table 1). Most of the reports of subcutaneous ticks are from red foxes, with occasional findings in other carnivore hosts (a raccoon dog, and a domestic dog) (Table 1). So far, several hypotheses have been suggested to explain the presence of ticks in subcutaneous tissue, but none of them has been confirmed by experimental studies. It is unknown if the number of reports and the relatively common occurrence of subcutaneous ticks in red foxes is related to host preference or to the number of studies performed on this wild carnivore species. Hence, elucidating the full host spectrum of subcutaneous ticks is important to fill in the knowledge gaps for this phenomenon. It is also unclear why most of the reports on subcutaneous ticks are from eastern and central Europe, and if this geographical bias is related to the fact that foxes are the most widespread and studied wild canids in this area. During the last 20 years, the population of another canid, the golden jackal (*Canis aureus*) has increased significantly [3]. Golden jackals have an important role as reservoir hosts for parasites such as *Leishmania infantum*, *Alaria alata*, *Dipylidium caninum*, *Mesocestoides lineatus*, *Trichinella* spp., and *Dirofilaria* spp., and are also hosts for ticks [4]. Moreover, it is not clear if other wild carnivores such as mustelids can harbor subcutaneous ticks, as the lack of published reports could be a result of the lack of investigations. The aim of the present study was to investigate the occurrence of subcutaneous ticks in various species of wild carnivores in a geographical area where this aberrant localization is known to be prevalent in red foxes, in order to elucidate the role of the host species.

**Table 1:** Review of reports of ticks in the subcutaneous tissues of various hosts

Host	Species	Country	Reference
Red fox *Vulpes vulpes*	*Ixodes ricinus* *Ixodes hexagonus*	UK	[12]
	*Ixodes ricinus*	Poland	[13]
	*Ixodes ricinus*	Austria	[14]
	*Ixodes ricinus*	Slovakia	[15]
	*Ixodes ricinus*	Slovakia	[16]
	*Amblyomma americanum*	USA	[17]
	*Ixodes ricinus* *Ixodes hexagonus* *Ixodes crenulatus* *Dermacentor reticulatus*	Czech Republic	[7]
	*Ixodes ricinus*	Romania	[7]
	*Ixodes ricinus*	Slovakia	[18]
	*Ixodes ricinus*	Sweden	[19]
	*Ixodes ricinus* *Dermacentor reticulatus*	Poland	[20]
	*Ixodes ricinus* *Ixodes hexagonus* *Ixodes canisuga*	Germany	[8]
Raccoon dog *Nyctereutes procyonoides*	*Ixodes ricinus*	Poland	[21]
Domestic dog *Canis familiaris*	*Ixodes ricinus*	Sweden	[19]
Golden jackal *Canis aureus*	*Ixodes ricinus*	Romania	Current study

## Materials and methods

Between 2015 and 2020, we necropsied 198 carcasses of 12 species of wild carnivores (57 golden jackals, six gray wolves, 19 wild cats, two Eurasian lynxes, 76 Eurasian badgers, 20 beech martens, eight European polecats, four European pine martens, three Eurasian otters, one stoat, one European mink, one least weasel) (Additional file 1) and examined them for parasites. The carcasses originated from roadkills or legally hunted animals. The carcasses were stored at −20 °C until processing. The age of the animals was estimated based on the state of tooth wear [5] and sexual maturity [6]. The carcasses were checked for the presence of ectoparasites, then necropsied using a standard method, starting with the removal of the skin. When subcutaneous ticks were found, the nodules were removed, carefully dissected, and stored in ethanol. The identification of the subcutaneous ticks was carried out to species level under an Olympus binocular magnifier and was based on taxonomic criteria according to dichotomous keys [1].

## Results

**Fig. 1:**
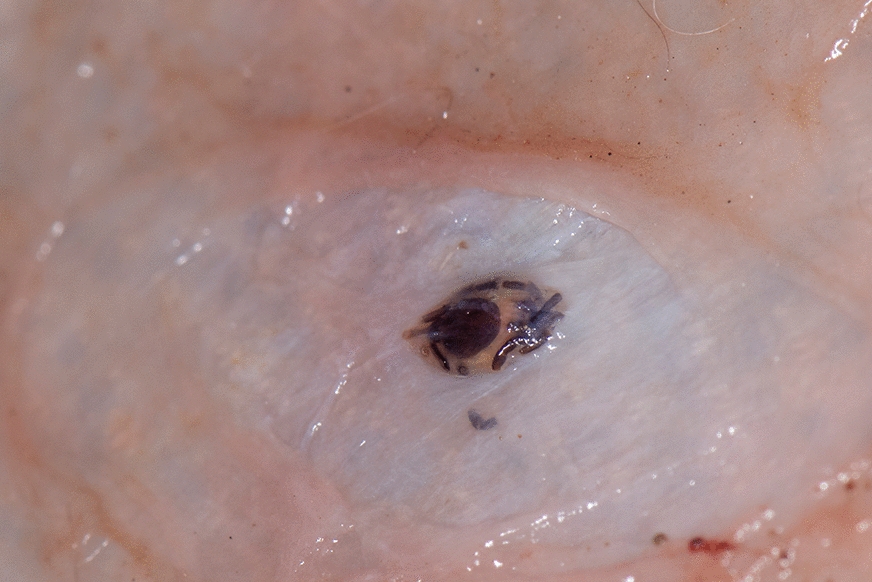
*Ixodes ricinus* in the subcutaneous tissue of a golden jackal *Canis aureus*, from Romania

A single subcutaneous tick was found in one sample, a golden jackal, collected from Comana Natural Park, Romania (Fig. 1). The nodule was found under the skin of the left inguinal area. The tick was in an advanced stage of decomposition. However, despite the level of tick degradation, the gnathosoma and a large part of the idiosoma were well preserved, and the tick was identified as a female *Ixodes ricinus*. No subcutaneous ticks were found in the other examined carcasses.

## Discussion

The mechanism causing the subcutaneous localization of ticks is still unknown, although several factors such as the species or the sex of the tick have been incriminated as favoring factors. A more common presence under the skin was noted for ticks with a long rostrum (i.e. *Ixodes* spp.) or that feed for longer periods (adults in general and females in particular), which seem to be predisposing factors [7, 8]. Although the vast majority of reports of subcutaneous ticks concern red foxes (Table 1), it is not clear if host-related factors are involved. The lack of reports of subcutaneous ticks in other hosts could be related to their absence in them or to the lack of studies on other hosts of ticks. To determine the full host spectrum of subcutaneous ticks, negative reports are also useful. However, with the exception of one study in roe deer [9], we know of no other negative reports.

To our knowledge, this is the first report of a subcutaneous tick in a golden jackal (frequency 1/57; 95 % confidence interval 0.04–9.39 %); subcutaneous ticks were absent from the other 11 species of carnivores examined. However, with the exception of a few host species such as Eurasian badger, beech marten and wild cat, the number of carcasses examined was too low for us to draw a firm conclusion from our results.

So far, with the exception of one human case, all reports of subcutaneous ticks are from studies on canids, with high local prevalence in red foxes [7, 8]. The vast majority of these reports refer to ticks of the genus *Ixodes* (Table 1), but this may be related to the more common occurrence of these ticks in red foxes [10, 11].

## Conclusion

We report a new host for subcutaneous ticks, and confirm that, to date, canids are the only group of carnivores to show this phenomenon. The results indicate a possible role of the host as a risk factor for subcutaneous ticks. We highlight the importance of carrying out further studies on other hosts of ticks, which should also be undertaken in other geographical regions.

## Supplementary information

**Additional file 1.** Database with all examined carnivores.

## Data Availability

The datasets used and/or analyzed during the current study are available from the corresponding author on reasonable request.
